# Hepatotoxicity induced by arsenic trioxide: clinical features, mechanisms, preventive and potential therapeutic strategies

**DOI:** 10.3389/fphar.2025.1536388

**Published:** 2025-02-20

**Authors:** Jun Wen, Aiwen Li, Ziliang Wang, Xiaoxiao Guo, Gaoling Zhang, Mark R. Litzow, Qiuju Liu

**Affiliations:** ^1^ Department of Haematology, Cancer Center, The First Hospital of Jilin University, Changchun, China; ^2^ Center of Hematology, Peking University People’s Hospital Qingdao, Qingdao, China; ^3^ Division of Hematology, Department of Medicine, Mayo Clinic, Rochester, MN, United States

**Keywords:** arsenic trioxide, hepatotoxicity, acute promyelocytic leukemia, natural small molecule extracts, reactive oxygen species

## Abstract

Arsenic trioxide (ATO) has shown substantial efficacy in the treatment of patients with acute promyelocytic leukemia, and the utilization of ATO as a potential treatment for other tumors is currently being investigated; thus, its clinical application is becoming more widespread. However, the toxicity of ATO has prevented many patients from receiving this highly beneficial treatment. The clinical features, mechanisms, and preventive measures for ATO hepatotoxicity, as well as potential curative strategies, are discussed in this review. This review not only discusses existing drugs for the treatment of hepatotoxicity but also focuses on potential future therapeutic agents, providing forward-looking guidance for the clinical use of small molecule extracts, trace elements, antidiabetic drugs, and vitamins.

## 1 Introduction

Arsenic has been used in traditional Chinese medicine for more than 2,000 years. In the 1970s, Chinese scholars first used ATO to treat acute promyelocytic leukemia (APL) and achieved significant therapeutic effects. ATO not only targets *PML/RARα* for degradation (Chen et al., 2011) but also promotes the differentiation of APL cells by specifically targeting zinc finger motif proteins in the RING and B1-box structural domains of PML (Breccia and Lo-Coco, 2012), leading to their degradation. In low concentrations, ATO induces the partial differentiation of cells, whereas at higher concentrations, it promotes apoptosis. Additional data suggest that ATO, but not All-trans-retinoic acid (ATRA), can eliminate leukemia-initiating zones in APL patients (Ablain and de The, 2011).

Single-agent ATO has been shown to induce profound molecular remission in patients with APL (Leu and Mohassel, 2009). Clinical trials, including CALGB C9710, have demonstrated that ATO-based regimens administered upfront offer superior outcomes compared to therapies that do not incorporate ATO (Powell et al., 2010). Further studies have reinforced these findings, highlighting excellent complete remission rates in patients treated with a combination of ATRA and ATO. As a result, the ATRA-ATO combination has become the standard of care for patients with low-to intermediate-risk APL (Lo-Coco et al., 2013). A randomized multi-center clinical trial, APL-15, demonstrated the feasibility and a high cure rate with ATRA and ATO treatment in patients with all-risk APL (Wang et al., 2022). For newly diagnosed patients with APL, the use of ATO, either alone or in combination with other drugs, can achieve a complete remission (CR) rate of over 90% (Leu and Mohassel, 2009; Powell et al., 2010; Lo-Coco et al., 2013; Wang et al., 2022), and 85% to 90% rates of 3 years survival (Powell et al., 2010). In patients with relapsed APL, reinduction can result in complete remission rates of more than 90% (Lou et al., 2015). Currently, ATO is considered one of the most efficacious drugs for the treatment of APL and has been designated the preferred option for treating newly diagnosed and relapsed APL by the National Comprehensive Cancer Network (NCCN) and other guidelines.

However, despite the remarkable therapeutic outcomes, increasing evidence suggests that the administration of ATO is not without its risks, particularly regarding liver toxicity. Increasing evidence indicates that the liver is not only the primary site for arsenic methylation but also a potential target for arsenic toxicity (Hu et al., 2018; Lu et al., 2019). The concentration of ATO in APL treatments exceeds the enforceable of WHO Maximum Contaminant Level (MCL) for arsenic of 0.01 mg/L (10 μg/L). When administered intravenously, it may exhibit significant bioaccumulation and more severe toxicity than drinking water contaminated with arsenic (Nithyananthan and Thirunavukkarasu, 2019). Long-term and high-dose ATO administration can lead to the bioaccumulation of arsenic metabolites, which can be retained at high concentrations during metabolism, resulting in liver injury (Liu et al., 2021). Patients with APL, who get ATO have been found to experience a higher rate of hepatotoxicity (Lo-Coco et al., 2013) than in those treated without ATO.

Since ATO is recognized as the extremely effective drugs for the treatment of APL, a better understanding of the underlying mechanisms of the liver toxicity induced by ATO are important for the development of specific and effective preventive measures. Currently, there is a lack of a systematic review of the literature on ATO-induced hepatotoxicity, with insufficient integration and summary of existing studies. Therefore, this review aims to provide a comprehensive analysis of recent research, systematically examining the clinical characteristics of hepatotoxicity, the various mechanisms by which ATO induces toxicity in the liver, and potential preventive strategies, offering a more holistic perspective and guiding future research directions.

## 2 Clinical features of arsenic hepatotoxicity

ATO can cause hepatotoxicity when administered both alone and in combination with other drugs. Studies have reported hepatotoxicity during APL treatment with ATO or in combination with ATRA, with an incidence ranging from 60% to 90% (Lo-Coco et al., 2013; Zhang et al., 2024; Mathews et al., 2006). Hepatotoxicity was most commonly observed during the initial 1–2 weeks of ATO treatment, with a median onset on day 6 (range, day 1–43). One early publication (Niu et al., 1999) noted that 7 (63.63%) of 11 newly diagnosed patients developed hepatotoxicity, and 2 of these patients failed to recover, with liver dysfunction contributing to their deaths. Mathews et al. (2006), and Mathews et al. (2010) reported that ATO causes liver toxicity when used alone to treat newly diagnosed APL patients. In their study of 76 patients, the incidence of hepatotoxicity was 38.2%, with grade (NCICTC v2.0) 1, 2, 3, and 4 liver injuries occurring in 18.4%, 9.2%, 5.3%, and 5.3% of patients, respectively. Hepatotoxicity occurred in 65.5% of patients during the induction phase, 10.3% during the consolidation phase, and 20.7% during the maintenance phase. One patient (3.4%) experienced hepatotoxicity after the completion of treatment.

The severity of ATO-induced hepatoxicity has changed over time. A previous study (Lo-Coco et al., 2013) demonstrated that 43 of 68 (63%) patients treated with ATRA–ATO had grade III or IV hepatic toxic effects (CTCAE) during induction, consolidation therapy, or maintenance therapy. In AML17 study of 109 patients, the incidence of hepatotoxicity was 69%, with grade (NCICTC v3.0) 1, 2, 3, and 4 liver injuries occurring in 23%, 23%, 20%, and 5% of patients, respectivelythe first course of treatment with ATO-ATRA (Burnett et al., 2015). Recently, in patients treated with ATO, Zhang et al. (2024) reported that hepatotoxicity continued to be a common toxicity that impacted therapeutic efficacy. According to the WHO toxicity grading scale for determining the severity of adverse events, the distribution of ATO-induced hepatotoxicity severity differed among their a total of 23.21% of patients with liver toxicity were grade III or IV. This study also indicated the severity of arsenic-induced hepatotoxicity was related to hepatoprotective medications. Table 1 summarizes the liver injury reported in the above literature.

**TABLE 1:** Statistics on the therapeutic occurrence of ATRA or combined with ATO.

Year	Patient (case)	Therapeutic occurrence of patient	Death	Treatment	Hepatotoxicity grading criteria	Specific grade	Ref.
1999	11 (Newly Diagnose)	7	2	ATO	NCICTC v1.0	2 cases in grade 1, 3 cases in grade 2, and 2 cases in grade 3	Moore et al. (2007)
	47 (Response)	15	0	ATO	NCICTC v1.0	14 cases in grade 1 and 1 case in grade 2)	Moore et al. (2007)
2010	72	24	0	ATO	NCICTC v2.0	19 patients in grade 1/2; 5 patients in Grade 3/4	Zacholski et al. (2022)
2023	122	112	0	ATO	WHO toxicity grading scale	51 cases were grade I toxicity, 35 cases were grade II toxicity	Mao et al. (2024)
						19 cases were grade III toxicity, and 7 cases were grade IV toxicity	
2012	68	43	0	ATRA + ATO	CTCAE	43 more than Grade 3–4	Powell et al. (2010)

ATO hepatotoxicity is a form of drug-induced liver injury (DILI) caused by antineoplastic drugs. The clinical manifestations of this DILI have not been found to be specific, and they start similarly to other acute and chronic liver disorders (Mao et al., 2024). Patients with acute-onset hepatocellular damage might not exhibit any symptoms in mild circumstances. In severe cases, patients may experience jaundice. Nonspecific gastrointestinal symptoms such as malaise, loss of appetite, anorexia, hepatic distension, and epigastric discomfort of varying degrees may also occur. Those with marked cholestasis may experience jaundice, light-colored stools, and pruritus. Patients who progress to acute hepatic failure/subacute hepatic failure (ALF/SALF) may present with jaundice, coagulation disorders, ascites, hepatic encephalopathy, and other related symptoms. Patients with special phenotypes may present with different clinical manifestations; for example, patients with drug hypersensitivity syndrome may exhibit extrahepatic symptoms such as fever and rash (Suk et al., 2012).

According to previous literature, ATO can contribute to hepatotoxicity based on three factors: host-dependent risk factors, underlying disease, and drug-dependent risk factors.

### 2.1 Host-dependent risk factors

The rate of severe adverse drug reactions (ADRs) increases with age (Moore et al., 2007). The physiological function of elderly patients declines, which slows the excretion of arsenic, leading to drug accumulation in the body and hepatotoxicity. Additionally, elderly patients often have chronic heart, liver, and kidney diseases, which exacerbate the accumulation of arsenic in the body and induce toxicity. Moreover, when ATO doses are administered based weight, high doses in overweight patients, a common occurrence in APL, may result in increased liver accumulation and hepatotoxicity (Zacholski et al., 2022).

### 2.2 Underlying diseases

Liver disease and differentiation syndromes may increase the risk of ATO-induced hepatotoxicity. Preexisting liver illness, such as chronic hepatitis B virus (HBV) or HCV infection, alcoholic liver disease, and high liver enzymes before the start of medication, were shown to be risk factors for DILI in one review (Núñez, 2006). The clinical manifestations of differentiation syndrome (Stahl and Tallman, 2019) include fever, dyspnea, hypoxemia, weight gain, pericardial effusion, postural hypotension, limb edema, and cardiac and renal insufficiency. These symptoms can lead to hepatocellular ischemia, hypoxia, and edema, which can further weaken hepatocellular function, slow the rate of arsenic elimination from the body, and ultimately exacerbate hepatotoxicity.

### 2.3 Drug-dependent risk factors

The dose of drug and hepatic drug metabolism are factors that can affect the level of hepatotoxicity. Long-term and high-dose administration of ATO can lead to the bioaccumulation of arsenic metabolites, which can be retained at high concentrations during metabolism, resulting in liver injury (Liu et al., 2021).

ATO is safe and effective within the clinical dose range. Daily low-dose ATO-induced hepatotoxicity can be mild and reversible and be alleviated after discontinuation or after hepatoprotective treatment (Wang et al., 2011). However, the rate of grade III/IV liver injury is still high, and death has been reported, with individual outcomes varying. Thus, regarding their clinical presentation, incidence of hepatotoxicity and risk factors, promptly monitoring and preventing ATO-induced hepatotoxicity is crucial to avoid serious consequences.

## 3 Arsenic metabolites

In the majority of preclinical and clinical studies, ATO was administered intravenously at a daily dose of 10 mg or 0.15 mg/kg/day, typically until complete remission was achieved or for a maximum duration of 60 days (Lo-Coco et al., 2013; Mathews et al., 2010; Burnett et al., 2015). The pharmacokinetic parameters of ATO in the bloodstream are of clinical significance and directly correlate with dosage (Liu et al., 2021). The mechanisms by which arsenic is metabolized in the body are as follows.

**FIGURE 1:**
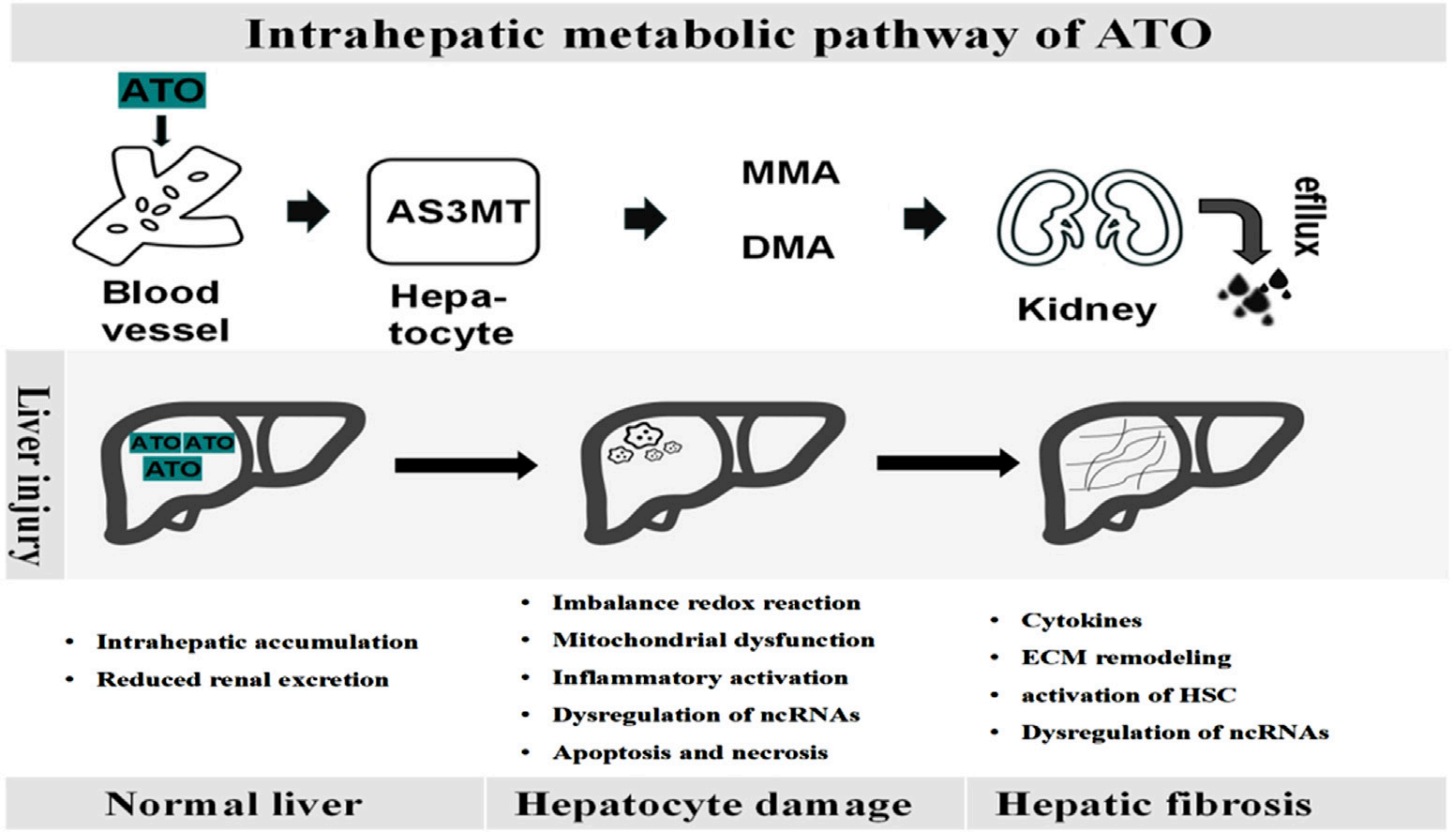
Dynamic pathological process of ATO-induced liver disease. Upon entering the bloodstream, ATO is metabolized in the liver by AS3MT into monomethylarsenate (MMA) and dimethylarsenate (DMA), which are then excreted through the kidneys. The accumulation of ATO within the liver leads to hepatocyte damage, parenchymal cell death, extracellular matrix deposition, and the activation of inflammatory pathways, ultimately contributing to progressive fibrosis.

After ingestion, ATO is absorbed into the bloodstream as arsenite (iAs^III^) and subsequently transported to the liver, where it undergoes methylation (Drobná et al., 2010). Hepatocytes uptake arsenate (As^V^) via phosphate transporter proteins and As^III^ through aquaglyceroporins. As^III^ is swiftly converted into trivalent active forms, such as monomethylarsonous acid (MMA^III^) and dimethylarsinous acid (DMA^III^), through a series of methylation reactions catalyzed by arsenic methyltransferase (AS3MT), in conjunction with S-adenosylmethionine (SAM) and glutathione (GSH) (Cullen, 2014; Dheeman et al., 2014). It is subsequently facilitated by arsenite efflux permease from the urine, most arsenic metabolites are excreted in the form of inorganic arsenic (iAs^V^) and the oxidized pentavalent forms of monomethyl arsenic (MMA^V^) and dimethyl arsenic (DMA^V^) (Ghiuzeli et al., 2022). Notably, research suggests that arsenic methylation may enhance its toxicity rather than promote detoxification (Hirano, 2020). The toxicity ranking of arsenic compounds is as follows: MMA^III^ > DMA^III^ > As^III^ > As^V^ > MMA^V^ > DMA^V^ (Raessler, 2018). As shown in Figure 1, arsenic metabolism processes *in vivo*.

## 4 Mechanisms of arsenic trioxide induced hepatotoxicity

**FIGURE 2:**
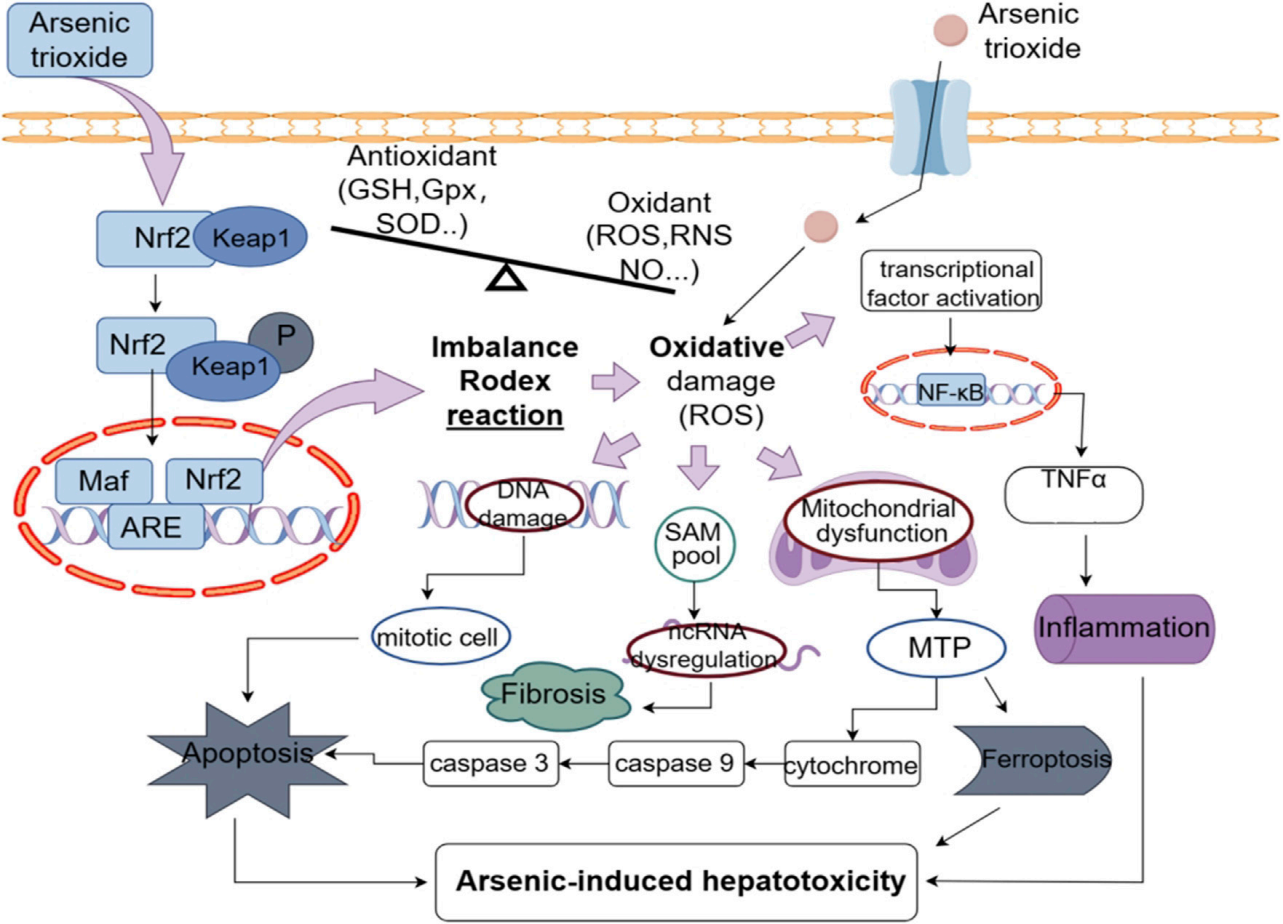
ATO leads to an increased production of ROS and thus to inflammation, apoptosis, fibrosis, ferroptosis, and further hepatotoxicity.

The relationship between oxidative stress and ATO-induced toxicity has been well established, although the specific molecular mechanism is still unclear. Arsenic induces oxidative stress by depleting antioxidants and elevating oxidant levels, leading to oxidative damage to DNA, lipids, and proteins (Xu et al., 2017), which is associated with the time-dependent generation of reactive oxygen species (ROS) (Santra et al., 2022). Compared to the low concentrations of iAsIII (less than 1 μM) in the blood of APL patients, MMAIII and DMAIII can generate reactive oxygen species (ROS) by targeting the mitochondria and endoplasmic reticulum (Naranmandura et al., 2011; Naranmandura et al., 2012a), thereby inducing cytotoxicity in normal cells. Research put forward that ATO-triggered oxidative stress by ROS can cause the activation of transcription factors, alter gene expression (Brunati et al., 2010; Adil et al., 2015; Ramachandran and Jaeschke, 2018) and activate autophagy, apoptosis, ferroptosis, inflammatory, fibrosis and necroptosis pathways (Chen et al., 2023). Li et al. (2015) reported that total antioxidative capacity (T-AOC) and GSH levels decreased, while MDA levels increased following ATO administration. Additionally, the serum ALT and AST levels significantly increased in the treatment group. Histopathological examination of the liver revealed changes in the structure and morphology of hepatocytes, thereby providing strong evidence that ATO induces liver damage through oxidative stress. Figure 2 shows how arsenic exposure damages the liver.

### 4.1 Nrf2 and oxidative stress

Nuclear factor erythroid 2-related factor 2 (Nrf2) is an important intracellular transcription factor and regulator of the antioxidant response, which is involved in ATO-induced oxidative liver injury (Yang et al., 2017a). Research has demonstrated that ATO induces hepatotoxicity through the Nrf2 signaling pathway by significantly reducing the overall Nrf2 content (Ghatak et al., 2011). Additionally, human juvenile hepatocytes exhibit reduced Nrf2 levels after 48 h of exposure to ATO (Vineetha et al., 2018). Furthermore, ATO reduces NQO1, GST, Bach-1 and HO-1 levels (Yang et al., 2017b), along with Nrf2 levels, which in turn leads to a diminished capacity to inhibit reactive ROS, ultimately compromising the efficacy of the antioxidant defense system against oxidative stress. These evidence suggest that ATO causes hepatotoxicity by generating excessive oxidative stress, which depletes Nrf2 and/or disrupts the equilibrium between Nrf2 production and degradation. This, in turn, impairs antioxidant defences, exacerbating the damage. However, the exact mechanism by which ATO regulates Nrf2 expression and its downstream effects on the antioxidant system remain unclear. Further research on this topic could be conducted in the future as a potential therapeutic strategy for the prevention or treatment of ATO-induced hepatotoxicity.

### 4.2 Cell death mechanisms

#### 4.2.1 Apoptosis

Arsenic induces cell Apoptosis by interacting with the permeability of mitochondrial membrane, opening the mitochondrial PTP, generating ROS, and releasing cytochrome c (Naranmandura et al., 2012b). Additionally, arsenic activates caspase-3 and caspase-9, leading to apoptosis (Sinha et al., 2013; Dong et al., 2020). Ahamed et al. discovered that arsenic exposure upregulated expression of the p53, Bax, caspase-3, and caspase-9 genes, downregulated Bcl-2 expression, and reduced the mitochondrial membrane potential, ultimately resulting in apoptosis (Ahamed et al., 2019). The review (Renu et al., 2020) summarises arsenic exposure induces apoptosis in liver tissue through various signaling pathways, including the AKT-PKB, PI3/AKT, MAPK, PKC δ-JNK, AKT/ERK, and p53 pathways.

#### 4.2.2 Autophagy

Studies indicate that ROS influence autophagy, which mediates ETosis brought on by ATO (Li et al., 2018). The induction of autophagy by ROS may result in the excessive self-digestion of cellular components and subsequent cell death. Goussetis et al. established that in addition to inducing apoptosis, ATO activates the MEK/ERK pathway to cause autophagy in leukemia cells, suggesting that autophagic cell death contributes to ATO suppression of leukemic hematopoiesis (Goussetis et al., 2010). Li et al. indicated that the formation of DNA traps known as ETosis, a unique cell death pathway that involves mTOR-mediated autophagy, can be induced by ATO in NB4 cells (Li et al., 2018).

#### 4.2.3 Ferroptosis

Ferroptosis is a a special type of programmed cell death that is triggered by lipid peroxidation and iron overload (Angeli et al., 2017). Xu et al. reported that arsenic exposure can increase ferrous ion and ROS levels, and by disrupting the balance between oxidation and antioxidation, increase the production of the lipid peroxidation product, malondialdehyde (MDA) (Xu et al., 2024). Glutathione peroxidase 4 (GPX4) and solute carrier family 7 member 11 (SLC7A11) are the key factors that promote ferroptosis (Yang et al., 2014). Arsenic, By triggering Nrf2-mediated adaptive antioxidant responses, can suppress the expression of GPX4, causing ferroptosis in healthy human hepatocytes and accelerating liver damage from arsenic exposure (Xu et al., 2024). Gao et al. demonstrated that ferroptosis is connected to liver damage caused by ATO (Gao et al., 2023).

Various cell death mechanisms, such as apoptosis, autophagy, and ferroptosis, are associated with ATO-induced hepatotoxicity; however, the relationship between these pathways has not been fully elucidated. How these mechanisms interact to contribute to ATO-induced liver injury, and whether there are potential synergistic effects, warrants further investigation. Targeting these pathways may hold potential for alleviating liver damage in patients undergoing ATO treatment and improving clinical outcomes.

### 4.3 Pathological changes in the liver caused by arsenic trioxide

#### 4.3.1 Inflammatory mechanism

Enhanced oxidative stress results in inflammation and the generation of pro-inflammatory cytokines, such as interleukin 6 (IL-6), interleukin 1β (IL-1β), and tumor necrosis factor-alpha (TNF-α), causing the disruption of the biological membrane (Liu et al., 2020). Li et al. reported that ATO increased the expression of the proteins IL-1β, IL-6, and TNF-α, enhancing inflammation (Li et al., 2020). Excessive ROS (Barchowsky et al., 1996) production can induce an inflammatory response mediated by nuclear factor-κB (NF-κB). When exposed to arsenite, hepatocytes activate proinflammatory NF-κB by increasing the phosphorylation and degradation of the inhibitor IκB, leading to elevated levels of TNF-α (Choudhury et al., 2016).

#### 4.3.2 Fibrosis mechanism

The networks of inflammation, cell death, and HSC activation mutually influence each other, resulting in initial hepatic steatosis and, with prolonged arsenic exposure, eventual portal fibrosis (Ghatak et al., 2011). Previous data indicate that prolonged exposure to arsenic results in oxidative stress, ROS production via the NADPH oxidase pathway, and increased Kupffer cell activation, leading to TNF-α production (Santra et al., 2000). This TNF-α production, in turn, leads to hepatocyte apoptosis and the activation of HSCs through the mitochondrial cytochrome c pathway. Activated HSCs produce type I collagen, as well as profibrotic PDGF and TGF-β, resulting in the upregulation of TIMP and MMP expression, which leads to extracellular matrix (ECM) remodeling and histological liver fibrosis (Ghatak et al., 2011). Dai J et al. results (Dai et al., 2020) show that ATO stimulates PML small ubiquitin-like modifier SUMOylation through the activation of TGF-β/Smad signaling pathway, up-regulating the production of inflammatory factors and activation markers in HSCs, eventually promoting the induction of liver fibrosis. However, the precise mechanisms underlying this SUMOylation process and its downstream effects on HSC activation and fibrosis remain unclear. It is crucial to investigate whether other post-translational modifications or signaling pathways may also contribute to ATO-induced liver fibrosis. Future studies focused on these unresolved aspects could significantly enhance our understanding of the fibrogenic mechanisms induced by arsenic exposure.

### 4.4 Epigenetic changes associated with dysregulated ncRNAs

Recent evidence suggests a correlation between arsenic-induced liver diseases and epigenetic alterations, particularly those linked to dysregulated non-coding RNAs (ncRNAs) (Li et al., 2021; Wu et al., 2023). NcRNAs, including microRNA (miRNA), long non-coding RNA (lncRNA), and circular RNA (circRNA), have been reported to play a role in arsenic-induced liver disease through dysregulation.

Arsenic metabolism has been shown to disrupt the regulation of ncRNAs. Previous studies have demonstrated that, on one hand, arsenic metabolism induces oxidative stress by disturbing redox homeostasis, resulting in increased reactive oxygen species (ROS) production and a diminished capacity of glutathione (GSH) to scavenge ROS. This imbalance subsequently promotes the biosynthesis of ncRNAs (He et al., 2014; Paul and Giri, 2015). On the other hand, arsenic metabolism leads to significant depletion of S-adenosylmethionine (SAM), which lowers the SAM pool and compromises genomic stability by altering DNA methylation patterns. This depletion further contributes to the dysregulation of ncRNAs. Moreover, alterations in the SAM pool influence m6A modification, a critical regulator of miRNA biosynthesis. This modification can affect both pre-miRNA processing and the splicing of arsenic-induced ncRNAs, leading to disrupted miRNA maturation and function (Han et al., 2021).

ATO upregulates the expression of miR-21 in LX-2 cells, while simultaneously downregulating PTEN expression. Additionally, it enhances the activation of recombinant human arginase 1, which subsequently fosters macrophage M2 polarization and the secretion of pro-fibrotic cytokines. These events collectively contribute to the development of liver fibrosis (Xue et al., 2021). The LncRNA HOTAIR contributes to arsenite-induced hepatic fibrosis by mediating dysregulated interactions between T cells and HSCs via miR-17-5p (Wu et al., 2023).

The impact of ATO on ncRNAs and their role in the progression of liver diseases through epigenetic modifications, such as DNA methylation and miRNA dysregulation, is promising but not yet well understood. Future studies exploring the relationship between ncRNAs and ATO-induced hepatotoxicity could unveil novel biomarkers or therapeutic targets.

## 5 Preventive and potential curative or curative strategies

### 5.1 Evaluation and dynamic monitoring

The occurrence of hepatotoxicity from ATO is related to the dose, duration of medication, and overall physical condition of the patient. Thus, evaluating patients’ liver function at the beginning of ATO use and monitoring it dynamically for patients with liver insufficiency is important. The most critical monitoring period is from 1 to 4 weeks after treatment. During ATO treatment, total protein, albumin, the albumin/globulin ratio, total bilirubin, prealbumin, and cholinesterase levels remained stable, while ALT, AST, and γ-GT levels temporarily increased. These findings imply that ATO-induced hepatotoxicity primarily involves acute injury and that dynamic monitoring of hepatic function indicators (Hao et al., 2013) is necessary during ATO therapy.

Other studies have shown that hemoglobin, white blood cell count, and fibrinogen can also be used to predict global ATO-induced hepatotoxicity independently (Zhang et al., 2024). Zheng Y et al. demonstrated that single nucleotide polymorphisms (SNPs) of the AS3MT gene ought to be considered when determining the therapeutic dose of ATO, and that the urinary primary methylation index (PMI) can be used as a monitoring indicator of ATO-related chronic adverse reactions (AEs) (Zheng et al., 2021). According to Sui et al. (2023), monitoring the levels of sulfhydryl compounds can indicate hepatic damage caused by ATO therapy, and hepatotoxicity can be assessed in advance. MiR-122 is a sensitive biomarker for the early prediction of liver injury, detectable before the elevation of ALT levels. As a hepatocyte-specific miRNA, it serves as a highly specific indicator of hepatic damage (Dear et al., 2018). An early prediction is beneficial to the patient, as it allows for a timely search for alternative treatments, more intensive monitoring, and the timing of the use of protective agents, among other things. Wang et al. indicated that peripheral blood mitochondrial DNA copy number (mtDNAcn) is anticipated to be a prospective biomarker of hepatotoxicity induced by ATO (Wang et al., 2023).

### 5.2 Hepatoprotective agents

Hepatoprotective agents are key therapeutic options for DILI, aiming to improve liver function, promote hepatocyte regeneration, and/or enhance hepatic detoxification. Regardless of their underlying mechanisms, these drugs can be broadly classified into two groups: those that primarily reduce ALT and/or AST levels, and those that target reductions in ALP and/or GGT levels (Mao et al., 2024). Magnesium isoglycyrrhizinate (MgIG) and bicyclic alcohol are commonly employed in clinical practice.

MgIG, one of glycyrrhizic acid preparations,is commonly used for the clinical treatment of DILI worldwide (Chen and Sun, 2011). MgIG can play a role of antioxidant, anti-inflammatory and hormone-like roles in DILI treatment and protect the liver against inflammatory damage. In a randomized, double-blind, multicenter Phase II trial, Wang and colleagues compared MgIG to tiopronin, a standard DILI therapy in China. The results demonstrated that intravenous administration of MgIG (200 mg/day for at least 2 weeks) effectively normalized ALT and AST levels in acute DILI patients (Wang et al., 2019).

Bicyclol is the first oral agent that was indicated for acute DILI and is registered for clinical trials. Its pharmacological effects primarily stem from its ability to suppress the expression and activity of inflammatory mediators triggered by liver injury, as well as reduce the production of ROS and nitric oxide (NO), thereby preserving antioxidants such as GSH (Liu et al., 2017). A multicenter, randomized, double-blind, double-dummy, active-controlled Phase II trial by Tang J et al. demonstrated that bicyclol exhibited promising efficacy and a favorable safety profile in managing acute DILI (Tang et al., 2022). These hepatoprotective drugs success in the treatment of DILI indicate that they could be potential candidates to protect patients from ATO-induced toxicity. Upon detecting ATO-induced hepatotoxicity, intravenous infusion of MgIG (Wang et al., 2019) (200 mg/day for at least 2 weeks) or oral bicyclol (Tang et al., 2022) (25 or 50 mg TID for 2–4 weeks) can be initiated. During this period, dynamic monitoring of the patient’s liver function should be conducted to determine whether an extension of treatment is necessary.

### 5.3 Potential curative strategies

#### 5.3.1 Potential antioxidants

Arsenic exposure can cause oxidative damage in the liver, leading to pathological changes at tapoptosis, inflammation, fibrosis. The favorable outcomes observed in basic studies substantiate the potential clinical therapeutic applications of these small-molecule antioxidant substances.

##### 5.3.1.1 Endogenous materials

Melatonin, a hormone generated by the pineal gland, has a significant protective effect against oxidative damage caused by external toxins. Zhang et al. (2017) demonstrated that melatonin mitigates ATO-induced hepatic damage by attenuating the accumulation of ROS and MDA, while simultaneously enhancing the activities of key antioxidant enzymes. Furthermore, melatonin alleviates ATO-induced liver injury through the activation of the Nrf2/HO-1 signaling pathway, mediated via the PI3K/AKT axis.

##### 5.3.1.2 Natural small molecule extracts

Supplementation with hydroxytyrosine-rich extract (Soni et al., 2020), diallyl triulfide (Miltonprabu and Sumedha, 2014), and tetrahydrocurcumin (Muthumani and Miltonprabu, 2015) alleviated arsenic-induced liver injury by restoring mitochondrial function. Mitochondrial ROS scavenging and enhanced antioxidant defense can be used to mitigate arsenic-induced liver injury. Ferulic acid has been demonstrated to mitigate arsenic-induced hepatotoxicity by lowering inflammation, decreasing oxidative stress, and suppressing the overexpression of PPAR-γ and GLUT2 proteins in the liver (Daryagasht et al., 2023). In rats exposed to arsenic, thymoquinone supplementation improves the liver’s overall metabolic and antioxidant state, reduces changes in hepatic arsenic accumulation, and lessens DNA damage in hepatocytes (Alam et al., 2022). Additionally, by scavenging ROS and decreasing arsenic-induced PINK1/Parkin pathway-mediated hepatic mitophagy, dictyophora polysaccharide exhibits hepatoprotective benefits (Hu et al., 2022).

Citicoline has been shown to enhance hepatic activity of key antioxidant enzymes, including catalase, superoxide dismutase, and glutathione peroxidase. Moreover, the administration of citicoline in sodium arsenite-intoxicated animals resulted in a significant reduction in the levels of caspase-3, TNF-α, IL-6 (Nikravesh et al., 2023). Chrysin reduces levels of PC, NO, MDA,TNF-α and IL-1β, decreasing the oxidative stress, reducing the inflammation and attenuating the histological lesions (Fatemi et al., 2021). Sulforaphane activated PI3K/Akt mediated Nrf2 signaling pathways to reduce oxidative stress and prevent liver toxicity (Thangapandiyan et al., 2019). Carnosic acid was found to reduce oxidative stress, inhibit MAPK activation, and attenuate apoptotic cell death pathways (Das et al., 2018). Other small molecular material mechanisms are shown in Table 2, such as dithiothreitol (Paul et al., 2008), crocin (Yousefsani et al., 2018), acety-L-carnitine (Bodaghi-Namileh et al., 2018), lutein (Li et al., 2015), pomegranate fruit extract (Choudhury et al., 2016), moringa oleifera leaf extract (Bashir et al., 2024), ginkgo biloba extract (Dong et al., 2021), gardenia latifolia extract (Mehboob et al., 2024), biochanin A (Jalaludeen et al., 2016), diosmin (Mirzaei et al., 2023), nobiletin (Ijaz et al., 2023).

**TABLE 2:** Summary of natural compounds used for inhibition Hepatotoxicity induced by Arsenic.

No.	Compound	Nature	Compound concentration	Animal model	Mechanism	References
1	Melatonin	Hormone	—	rats	Decreasesd the levels of ROS and MDA and activated PI3K/AKT pathway	Ijaz et al. (2023)
2	Dithiothreitol	dithiol compound	—	Male Sprague Dawley (SD) rats	Regulated the mitochondrial permeability transition and membrane depolarization potential to prevent cell death in Hep 3B cells	Mouchel et al. (2023)
3	Crocin	Carotenoid compound	pretreatment with crocin at a concentration of 25 μg/mL	Male Sprague-Dawley rats	Inhibited ROS generation, GSH oxidation, and cytochrome c release from mitochondria	Binu et al. (2018)
4	Acetyl-L-carnitine	Dietary supplement	100,200/300 mg/kg/day for 21 days	male Wistar rats	Inhibited ROS generation, GSH oxidation, and cytochrome c release from mitochondria	Zhang et al. (2014)
5	Lutein	Dietary supplement	40 mg/kg/day for 5 weeks	male and female, Kunming mice	Improved the activities of antioxidant enzymes, attenuated increasing of ROS and MDA induced by arsenic trioxideandincrease the mRNA and protein expression of Nrf2 signaling related genes (Nrf2, Nqo1, Ho-1, and Gst)	Chen et al. (2023)
6	Ferulic acid	Plant extract-phenolic	100 mg/kg/day for 30 days	male NMRI mice	Improved the activities of antioxidant enzymes, attenuated increasing of ROS and MDA induced by arsenic trioxide and increase the mRNA and protein expression of Nrf2 signaling related genes (Nrf2, Nqo1, Ho-1, and Gst)	Ahangarpour et al. (2017)
7	Diosmin	Plant extract-polyphenolic phytochemicals	10 mg/kg/day for 4 weeks	male NMRI mice	Diminished the level of nitric oxide, tumor necrosis factor-alpha, protein amount of Sirtuin 3 and nuclear factor kappa B, and thiobarbituric acid reactive substances; increased total thiol and enzymatic activities of catalase, superoxide dismutase, and glutathione peroxidase in liver tissue	Abdel-Wahab et al. (2024)
8	Nobiletin	Plant extract-polyphenolic phytochemicals	25 mg/kg/day for 30 days	Sprague Dawley rats	Increased the antioxidant enzyme activity, with a noteworthy reduction in the deposition of As in hepatic tissues, TBARS, and H2O2 levels	Messarah et al. (2012)
9	Dictyophora Polysaccharide	Plant extract-Polysaccharide	80 μg/mL for 4 h	Normal human liver cell line L-02	Reduced As-induced PINK1/Parkin pathway-mediated hepatic mitophagy through scavenging ROS and exert hepatoprotective effects	Pilsner et al. (2011)
10	Thymoquinone	Plant extract	1.5 mg/kg bwt for 30 days	Male NIB Wistar rats	Upregulate the transcription of SOD, CAT, and GSH-Px genes and elevate the activities of these enzymes in the rat liver ; improved the overall hepatic metabolic and antioxidant status	Ren et al. (2021)
11	Citicoline	Plant extract	250/500/1,000 mg/kg/day 2 weeks	male NMRI mice	Increased the hepatic activity of catalase, superoxide dismutase, and glutathione peroxidase enzymes; reduced levels of caspase 3, tumor necrosis factor-alpha, and interleukin 6	Smits et al. (2019)
12	Chrysin	Plant extract-polyphenolic phytochemicals	25/50/100 mg/kg/day, 21 days	male Wistar rats	Reduced PC, NO and MDA,TNF-α and IL-1β levels; decreasing the oxidative stress, reducing the inflammation and attenuating the histological lesions	Ozoani et al. (2024)
13	Sulforaphane	Plant extract	20/40/80 mg/kg BW for 4 weeeks	Male albino Wistar rats	Activated PI3K/Akt mediated Nrf2 signaling pathways to Reduce Oxidative Stress and prevent liver toxicity	Vineetha et al. (2018)
14	Carnosic Acid	Plant extract-phenolic diterpene	1/2/4/6/10 μM in rat liver cell	Male Swiss albino mice	ReducED oxidative stress, MAPK activation, and apoptotic cell death pathway	Mathews et al. (2006)
15	Biochanin A	Plant extract	10/20/40 mg/kg/day	rats	Free radical scavenging and membrane stabilizing properties to counteract oxidative damage	Sankar et al. (2015)
16	Pomegranate fruit extract	Plant extract	2.7 mg/kg/day for 30 days	male Swiss albino mice	Reversal of ROS-dependent apoptosis and the mitochondrial membrane potential in hepatocytes	Paul and Giri (2015)
17	Moringa oleifera Leaf Extract	Plant extract	100/150 mg/kg/day for 3 months	Sprague Dawley rats	Improved the liver’s cellular integrity, correcting liver proteins/enzymes and inhibiting TBARS levels by activating the antioxidant enzymes in a dose-dependent manner	Bashir et al. (2024)
18	Gardenia latifolia Extract	Plant extract	200 mg/kg/d for 4 weeks	male albino rats from IMBB, UOL animal house	Decreased scavenging activities of H2O2, nitric oxide, superoxide, and DPPH, reducing oxidative stress and enhancing the antiproliferative effectiveness	Mehboob et al. (2024)
19	Ginkgo biloba extract	Plant extract	50 mg/kg for 6 weeks	male and female Wistar rats	Suppressed the overactivated inflammatory-related TLR4-MyD88-NF-κB pathway and evidently decreased the secretion of inflammation cytokines	Dong et al. (2021)
20	L-Ascorbic acid (L-AA)	Dietary supplement	25 mg/100 g/qod for 30 days	male Wistar rats	Inhibited ROS generation,GSH oxidation and improving the structure and function of liver mitochondria	Mondal et al. (2016)
22	Eugenol	Plant extract	5 mg/kg bwt for 30 days	male Wistar rats	Reduced the lipid peroxidation rate and arsenic deposition	Binu et al. (2018)
23	Resveratrol	Plant extract	3 mL/kg/qod for 3 days	male and female Chinese Dragon-Li cats	Inhibited ROS generation and increased SOD and CAT activity, as well as the GSH/GSSG ratio	Zhang et al. (2014)

Many natural small molecule extracts exhibit good antioxidant effects and may play a beneficial role in protecting against ATO-induced liver injury. However, the standardization of plant-derived compounds may pose a challenge, as natural extracts often vary in the concentration of active ingredients due to differences in cultivation conditions, and the bioavailability in humans can significantly differ from that in animal models.

##### 5.3.1.3 Vitamins

Vitamin C (L-AA) and vitamin E (α-tocopherol) are commonly used for their antioxidant effects. In the treatment of APL, vitamins are often used to reduce the toxicity of ATO. L-Ascorbic acid not only enhanced the antitumor effect of ATO but also reduced ATO-induced liver damage in rats (Singh and Rana, 2010).

Studies point out that the combination of L-AA and ATO can improve the structure and function of liver mitochondria. L-AA can significantly increase the ratio of ADP:O in mitochondria, restore the activities of succinate dehydrogenase and ATPase, reduce the activity of the hepatic caspase-3 enzyme, and decrease the percentage of apoptotic hepatocytes. The main mechanism of its hepatoprotective effect is its antioxidant activity. L-AA inhibits hepatic lipid peroxidation, regulates GSH levels, and restores hepatic glutathione S-transferase activity.

Furthermore, the analog of α-tocopherol, water-soluble vitamin E (Trolox), has been shown to reduce the hepatotoxicity of ATO while enhancing its anti-lymphoma effects. Additionally, folate, vitamin B12, and phosphate alleviate hepatic mitochondrial dysfunction by reducing free radical generation and lipid peroxidation while enhancing the antioxidant defense system (Majumdar et al., 2011).

The combination of L-AA and vitamin E reduces the cytotoxicity of ATO to human liver cells *in vitro*. This combination also inhibits lipid peroxidation and restores the activity of antioxidant enzymes and the potential of mitochondrial membranes. These effects are related to upregulated expression of the antioxidant protein Bcl-2 by Nrf2. *In vivo*, the combination of these two substances reduced the levels of MDA and thiobarbituric acid reactants and restored GSH levels in the liver of rats treated with ATO (Mondal et al., 2016).

Current studies also indicate that high doses of Vitamin C have antitumor effects, and related clinical trials are underway (Mouchel et al., 2023). However, there is still a lack of data on its use as adjunctive therapy for ATO-induced liver toxicity. Further clinical trials are needed to assess the optimal dosage, potential side effects, and long-term effects.

##### 5.3.1.4 Ingredients in Chinese medicine

Chinese medicine is a valuable cultural heritage of the Chinese nation and an essential component of traditional medicine worldwide. The hepatotoxicity induced by ATO is caused by a variety of pathological mechanisms as we have described. The subsequent screening of active ingredients in traditional Chinese medicine for joint use with ATO will enhance the antitumor effect of ATO and expand the scope of its clinical application, while also reducing its toxicity and side-effects. This will facilitate the use of ATO in a more extensive and safer manner.

Eugenoln is a monoterpene found in clove oil that is used in Chinese medicine for the treatment of hypertension and local analgesia. According to Binu et al. (2018), in rats’ liver tissue, eugenol administration decreased the rate of lipid peroxidation and arsenic deposition while also improving antioxidant status.

Resveratrol is a nonflavonoid polyphenolic compound that is found in grape skin, peanuts, and traditional Chinese medicines such as He Shouwu, Tiger Balm, Mulberry, and other plants. Modern pharmacological studies have shown that it has anti-inflammatory, antioxidant, anti-aging, platelet aggregation inhibition, immunomodulatory, and antitumor effects. Zhang et al. (2014) reported that coadministration of ATO with resveratrol significantly increased SOD and CAT activity, as well as the GSH/GSSG ratio, in rats. Additionally, the ROS level in the liver decreased, and arsenic accumulation decreased. These results suggest that resveratrol can attenuate ATO-induced hepatotoxicity by reducing oxidative stress and arsenic accumulation in the liver.

##### 5.3.1.5 Hypoglycemic drugs

Metformin and dapagliflozin have been demonstrated to play a significant role in the reduction of glucose levels. However, recent basic studies have indicated that they also possess considerable potential in the mitigation of ATO-induced hepatotoxicity.

Metformin, a first-line oral antidiabetic agent, has also been reported to alleviate hepatotoxicity induced by ATO (Ling et al., 2017) and sodium arsenite-induced hepatic mitochondrial toxicity (Ahangarpour et al., 2017) by modulating mitochondrial function.

*Dapagliflozin* (Abdel-Wahab et al., 2024) regulates autophagy by inhibiting the PI3K/Akt/mTOR signaling pathway. This inhibition reduces inflammation and oxidative stress in hepatocytes by regulating the PI3K/Akt/mTOR and STAT3/SOCS3/p53/MDM2 signaling pathways and miRNA-21 and miRNA-122 expression. A mouse model showed restored serum and tissue biomarker levels, improved liver function, and attenuated ATO-induced hepatotoxicity.

Both drugs are currently in clinical use for the treatment of diabetes, but their role in treating arsenic-induced liver damage would need to undergo rigorous clinical validation. For instance, metformin is associated with known side effects (e.g., gastrointestinal discomfort, lactic acidosis), which could complicate its use in patients with liver damage. Similarly, the dual action of dapagliflozin on both liver function and glucose levels would necessitate extensive clinical trials to evaluate its long-term safety in populations with liver dysfunction.

##### 5.3.1.6 Trace elements

Selenium (Se) and zinc are essential trace elements vital for normal growth and overall health in both humans and animals. Previous research has highlighted the protective effects of therapeutic doses of Se against arsenic induced liver injury (Messarah et al., 2012). Specifically, one study demonstrated that selenium, when administered within safe dosage ranges, mitigated arsenic-induced hepatic damage in chickens through antioxidant mechanisms and enhanced biliary excretion (Ren et al., 2021). Pilsner et al. reported findings indicating that Se facilitated the reduction of blood concentrations of the arsenic metabolite, MMA (Pilsner et al., 2011). Additionally, controlled experiments of Bangladesh revealed that a high arsenic lentil diet led to an increase in urinary arsenic excretion (Smits et al., 2019). According to Harrison Ozoani et al., zinc and selenium both reduced arsenic-induced hepatotoxicity, due to the inhibition of inflammo-oxidant signaling pathways (Ozoani et al., 2024). While selenium and zinc supplementation have shown promise, their use in humans needs careful consideration due to the narrow therapeutic index of these elements. Clinical trials would need to evaluate the safety of these elements over long periods, including potential interactions with other treatments and the appropriate dosage for different demographic groups.

#### 5.3.2 Nanoencapsulation technology

Nanoparticle-loaded drugs represent a common technique employed in drug delivery systems, with the objective of enhancing drug solubility, targeting and bioavailability. Mondal et al. demonstrated that Morin encapsulated in chitosan nanoparticles (MCNPs) exhibits superior hepatoprotective effects compared to free Morin, particularly in mitigating arsenic-induced toxicity. This protective action is attributed to the potent antioxidant, anti-apoptotic, and anti-inflammatory properties of the MCNP formulation (Mondal et al., 2022). Sankar et al. reported that a nanoparticle-encapsulated curcumin formulation offers enhanced efficacy over free curcumin in combating arsenic-induced hepatic oxidative stress in rats (Sankar et al., 2015). But we need to be vigilant that nanoparticles may have unique toxicological profiles not seen in conventional drugs, and their interactions with biological systems may lead to unintended side effects. Long-term safety studies would be crucial.

## 6 Conclusion and prospects

ATO has proven effective in the clinical management of acute promyelocytic leukemia and has shown promising potential in the treatment of various other tumors and diseases. However, the hepatotoxicity associated with ATO necessitates urgent attention. This review offers valuable insights into the mechanisms underlying ATO-induced apoptosis, inflammation, fibrosis, and provides a systematic understanding of how to prevent, monitor, and treat arsenic hepatotoxicity. In addition, it explores the therapeutic challenges, including the resistance to numerous antioxidants still under investigation and the use of hepatoprotective agents commonly employed in clinical settings.

NcRNAs are implicated in the general mechanism of arsenic-induced liver disease and may be attractive tools and targets for new therapeutic approaches. In the future, the role of ncRNAs in arsenic-induced liver diseases should be fully explored in order to identify precise targets for the prevention of ATO hepatotoxicity. Furthermore, several studies have shown that dysregulation of m6A regulatory factors plays a crucial role in leukemia, and alterations in the mRNAs they target (such as METTL14 and FTO) may be associated with the development of drug resistance [Yan et al., 2018; Weng et al., 2024; Hsu et al., 2017]. In the future, we could further expand on this by investigating the relationship between m6A modifications of ncRNAs and ATO treatment resistance. The exploration of phytochemicals, natural compounds, and targeted nutritional formulations that not only mitigate arsenic-induced toxicity but also act as therapeutic agents to reverse its harmful effects remains a highly promising area of research. Moreover, further comprehensive investigations into the utilization of nanodrug carriers may reduce arsenic-induced hepatotoxicity. However, these studies are currently in the preclinical stage, necessitating further clinical trials to establish their safety and therapeutic efficacy.
